# mBISON: Finding miRNA target over-representation in gene lists from ChIP-sequencing data

**DOI:** 10.1186/s13104-015-1118-8

**Published:** 2015-04-16

**Authors:** Marie Luise Gebhardt, Arvind Singh Mer, Miguel Angel Andrade-Navarro

**Affiliations:** Max Delbrück Center for Molecular Medicine, Berlin, 13125 Germany; Institute of Molecular Biology, Mainz, 55128 Germany; Faculty of Biology, Johannes-Gutenberg University of Mainz, Mainz, 55128 Germany; Department of Medical Epidemiology and Biostatistics, Karolinska Institute, Stockholm, Sweden

**Keywords:** microRNA, ChIP-sequencing, Enrichment, Target genes, Gene regulatory networks, Transcription factors, Data integration

## Abstract

**Background:**

Over-representation of predicted miRNA targets in sets of genes regulated by a given transcription factor (e.g. as defined by ChIP-sequencing experiments) helps to identify biologically relevant miRNA targets and is useful to get insight into post-transcriptional regulation.

**Findings:**

To facilitate the application of this approach we have created the mBISON web-application. mBISON calculates the significance of over-representation of miRNA targets in a given non-ranked gene set. The gene set can be specified either by a list of genes or by one or more ChIP-seq datasets followed by a user-defined peak-gene association procedure. mBISON is based on predictions from TargetScan and uses a randomization step to calculate False-Discovery-Rates for each miRNA, including a correction for gene set specific properties such as 3’UTR length. The tool can be accessed from the following web-resource: http://cbdm.mdc-berlin.de/~mgebhardt/cgi-bin/mbison/home.

**Conclusion:**

mBISON is a web-application that helps to extract functional information about miRNAs from gene lists, which is in contrast to comparable applications easy to use by everyone and can be applied on ChIP-seq data directly.

## Findings

It has been demonstrated that sets of functionally related genes, e.g. genes from a protein complex [1] or sets regulated by a common transcription factor [2,3], may contain information about their regulation on post-transcriptional level, which can be uncovered by means of enrichment analysis of miRNA targets.

An application of such enrichment analysis can facilitate the classification of predicted miRNA targets according to their likelihood of being biologically functional and can point to miRNA function [2].

Considering that a reliable experimental assignment of targets to miRNAs in large scale is still very challenging, it is desirable to take advantage of the growing amounts of ChIP-seq data that are deposited in databases like GEO [4].

The mBISON (miRNA binding site over-representation) tool was developed to enable the direct use of gene lists or ChIP-seq data to address the above mentioned questions. It takes a very simple input and applies a fast simulation approach to calculate False-Discovery-Rates (FDRs) for over-representation of miRNA targets. The results are corrected taking into account specific properties of the gene set that could bias the outcome.

### Tool description

There are two ways to use the web-application:

1. Enter or upload a gene list. The user can choose from different identifiers (Entrez-ID, Gene Symbol, Ensembl ID or RefSeq-ID); the recommended input is Entrez-IDs.
2. Upload one to three ChIP-seq datasets in bed-format supplying genomic positions of e.g. transcription factor binding sites (TFBSs) of the master factor to the “Peak-gene association” section of the webpage. The tool will analyze the data to assign TFBSs to genes as defined in RefSeq [5]. Assigning peaks to genes can be done in different ways. The user can choose either to look for genes nearest to the peaks (in range of 5, 10 or 20 kb off the transcription start site of a gene) or to use the ranked peak-gene association method, which is based on the idea that transcription factor binding can often be found either in the core promoter region or in the first intron of a gene ([6]; see (Gebhardt et al.[2]) for more details). If more than one bed-file is uploaded only genes having at least two times a peak in proximity will be considered. Subsequently the list will be analyzed by the mBISON tool for over-representation of predicted miRNA-targets.

mBISON is based on the conserved miRNA binding site predictions of TargetScan 6.2 with restriction to (broadly-)conserved miRNA-families to ensure the use of high quality predictions. Human or mouse gene sets can be analyzed [7]. Predictions for all isoforms of a gene were pooled. To create a final dataset for simulation (background) all possible unique miRNA-target gene pairs were collected (see [2] for details).

mBISON will check how many genes *N* from the input gene set can be found in the TargetScan background, since not all genes have predicted miRNA binding sites in the 3′UTRs. Genes without predicted binding sites will be excluded from the analysis. The tool will run if *N* is between 20 and 4000 genes. The upper bound is necessary due to computational limitations; nevertheless, transcription factors binding to too many places in the genome cannot be expected to give significant enrichment results. The user can specify the FDR that he regards as reasonable cutoff between 0.2 and 0.005. A second cutoff can be set, which introduces the minimum number of required targets for each miRNA-family as percentage of *N*.

Taking the gene list as input mBISON outputs one FDR (of over-representation in the 3′UTRs of the respective genes) for each of 153 miRNA-families. The FDR for a miRNA-family miR-A is calculated by checking if the number *m*_*A*_ of predicted targets in the gene set is larger than the count of predicted targets *z*_*A*_ of a random gene set chosen from the background. It is very important to take properties of the input gene set into account to avoid biases. For example, if the gene set had on average longer 3′UTRs than the background, more targets would be predicted for each miRNA and too many miRNA-families would appear significantly over-represented. To take properties of the input gene set into account *z*_*A*_ is multiplied by the ratio of total predicted targets for all 153 miRNAs in the gene set to the total predicted targets for the random set (see [2] for details). Repeating this procedure 1,000, 10,000 or 100,000 times results in a p-value for miR-A, which is corrected for multiple testing by the Benjamini and Hochberg method.

If the user provides the identifier of the master factor regulating the gene set, mBISON will point to miRNA-families that are predicted to regulate the master. Over-represented miRNAs that target both the master and the gene set assemble a coherent or incoherent feedforward loop of type 2 [8]. The tool will moreover help the user to identify negative feedback loops by listing miRNAs that are targeted by the master (miRNA-genes with a peak close by, distance of 5, 10 or 20 kb, according to miRBase, release 20 [9]).

The mBISON output can be downloaded as text-file. All miRNA-gene pairs from the gene set and over-represented miRNA-families are made available in a separate text-file. This is useful if the user wants to perform subsequent analysis on the targets of an over-represented miRNA (e.g. Gene Ontology enrichment analysis) or is interested in specific target genes.

### Example

We uploaded a bed-file containing beta-catenin binding regions in SW480 colorectal cancer cells (GSE53927 in GEO [4]) to mBISON and found miR-183 to be the top-enriched miRNA in this context. This miRNA is known to be positively regulated by beta-catenin directly in human gastric cancer [10] and to inhibit the Wnt/beta-catenin pathway in turn by targeting LRP6 in 3 T3-L1 cells [11].

## Conclusion

Most tools that make use of enrichment of miRNA targets involve functional annotation databases (e.g. Gene Ontology or KEGG pathways) and are not designed to look for pure over-representation of miRNA targets in gene lists [12]. miTEA is to our knowledge the only web-application that searches for enrichment of miRNA targets, but it needs a ranked gene list as input, which is usually obtained with the help of miRNA or gene expression data [13]. It can therefore not be easily applied to ChIP-seq data. MirBridge is a sophisticated algorithm for detection of miRNA target enrichment (not available online) that makes use of a simulation taking properties of the input gene list by means of GC content and general conservation into account [1]. It provides results of high quality but the underlying algorithms rely on multiple simulations that cause long runtimes and make it unsuitable for a web-application. The mBISON web-application fills a gap here.

We note that while some master factors might be part of a regulatory network involving many miRNAs and could show significant results, as in the case of REST [2], other factors might not have a single enriched miRNA-family.

By definition, miRNA-families identified as over-represented by mBISON target a significant fraction of the input gene set and may indicate that the miRNA has a function similar to the one of the master regulator. Thus, mBISON not only points to miRNA targets with increased likelihood of biological functionality but also allows to some degree functional annotation of miRNAs; this can be helpful in any miRNA-related field. Hypotheses and suggested relations might help to develop reasonable experimental setups to explore the respective biological system. The web-application can easily be applied by users without experience in bioinformatics.

### Availability and requirements

**Project name:** mBISON

**Project home page:**http://cbdm.mdc-berlin.de/~mgebhardt/cgi-bin/mbison/home

**Operating system:** platform independent

**Requirements:** browser

### Availability of supporting data

The dataset supporting the results of this article is available in the NCBI GEO repository, [GSE53927, http://www.ncbi.nlm.nih.gov/geo/query/acc.cgi?acc=GSE53927].

## Abbreviations

miRNA: MicroRNA
ChIP-seq: ChIP-sequencing
mBISON: MiRNA binding site over-representation
TFBS: Transcription factor binding site
FDR: False-discovery-rate
ID: Identifier
3′UTR: 3-prime untranslated region
